# Discordant bioinformatic predictions of antimicrobial resistance from whole-genome sequencing data of bacterial isolates: an inter-laboratory study

**DOI:** 10.1099/mgen.0.000335

**Published:** 2020-02-12

**Authors:** Ronan M. Doyle, Denise M. O'Sullivan, Sean D. Aller, Sebastian Bruchmann, Taane Clark, Andreu Coello Pelegrin, Martin Cormican, Ernest Diez Benavente, Matthew J. Ellington, Elaine McGrath, Yair Motro, Thi Phuong Thuy Nguyen, Jody Phelan, Liam P. Shaw, Richard A. Stabler, Alex van Belkum, Lucy van Dorp, Neil Woodford, Jacob Moran-Gilad, Jim F. Huggett, Kathryn A. Harris

**Affiliations:** ^1^​ Clinical Research Department, London School of Hygiene and Tropical Medicine, London, UK; ^2^​ Microbiology Department, Great Ormond Street Hospital NHS Foundation Trust, London, UK; ^3^​ Molecular and Cell Biology Team, National Measurement Laboratory, Queens Road, Teddington, Middlesex, UK; ^4^​ Institute for Infection and Immunity, St George’s, University of London, Cranmer Terrace, London, UK; ^5^​ Pathogen Genomics, Wellcome Sanger Institute, Wellcome Genome Campus, Hinxton, UK; ^6^​ Department of Infection Biology, London School of Hygiene and Tropical Medicine, London, UK; ^7^​ Clinical Unit, bioMérieux, La Balme Les Grottes, France; ^8^​ Vaccine and Infectious Disease Institute, Laboratory of Medical Microbiology, Faculty of Medicine and Health Sciences, University of Antwerp, Antwerp, Belgium; ^9^​ National University of Ireland Galway, Galway, Ireland; ^10^​ NIS Laboratories, National Infection Service, Public Health England, London, UK; ^11^​ Carbapenemase-Producing Enterobacterales Reference Laboratory, Department of Medical Microbiology, University Hospital Galway, Galway, Ireland; ^12^​ School of Public Health, Faculty of Health Sciences, Ben-Gurion University of the Negev, Beer Sheva, Israel; ^13^​ Department of BiNano Technology, College of BiNano Technology, Gachon University, Seoul, Republic of Korea; ^14^​ Nuffield Department of Medicine, University of Oxford, John Radcliffe Hospital, Oxford, UK; ^15^​ AMR Centre, London School of Hygiene and Tropical Medicine, London, UK; ^16^​ UCL Genetics Institute, Department of Genetics, Evolution and Environment, University College London, Gower Street, London, UK; ^17^​ School of Biosciences and Medicine, Faculty of Health and Medical Science, University of Surrey, Guildford, UK

**Keywords:** antimicrobial resistance, antimicrobial-susceptibility testing, whole-genome sequencing, bioinformatics, carbapenem resistance

## Abstract

Antimicrobial resistance (AMR) poses a threat to public health. Clinical microbiology laboratories typically rely on culturing bacteria for antimicrobial-susceptibility testing (AST). As the implementation costs and technical barriers fall, whole-genome sequencing (WGS) has emerged as a ‘one-stop’ test for epidemiological and predictive AST results. Few published comparisons exist for the myriad analytical pipelines used for predicting AMR. To address this, we performed an inter-laboratory study providing sets of participating researchers with identical short-read WGS data from clinical isolates, allowing us to assess the reproducibility of the bioinformatic prediction of AMR between participants, and identify problem cases and factors that lead to discordant results. We produced ten WGS datasets of varying quality from cultured carbapenem-resistant organisms obtained from clinical samples sequenced on either an Illumina NextSeq or HiSeq instrument. Nine participating teams (‘participants’) were provided these sequence data without any other contextual information. Each participant used their choice of pipeline to determine the species, the presence of resistance-associated genes, and to predict susceptibility or resistance to amikacin, gentamicin, ciprofloxacin and cefotaxime. We found participants predicted different numbers of AMR-associated genes and different gene variants from the same clinical samples. The quality of the sequence data, choice of bioinformatic pipeline and interpretation of the results all contributed to discordance between participants. Although much of the inaccurate gene variant annotation did not affect genotypic resistance predictions, we observed low specificity when compared to phenotypic AST results, but this improved in samples with higher read depths. Had the results been used to predict AST and guide treatment, a different antibiotic would have been recommended for each isolate by at least one participant. These challenges, at the final analytical stage of using WGS to predict AMR, suggest the need for refinements when using this technology in clinical settings. Comprehensive public resistance sequence databases, full recommendations on sequence data quality and standardization in the comparisons between genotype and resistance phenotypes will all play a fundamental role in the successful implementation of AST prediction using WGS in clinical microbiology laboratories.

## Data Summary

Sequence read files for all samples used in this study have been deposited in the European Nucleotide Archive under the project accession number PRJEB34513 and the following sample accession numbers: SAMEA5789893 (sample A-1), SAMEA5789894 (sample A-2), SAMEA5789895 (sample B-1), SAMEA5789896 (sample B-2), SAMEA5789897 (sample C-1), SAMEA5789898 (sample C-2), SAMEA5789899 (sample D), SAMEA5789900 (sample E), SAMEA5789901 (sample F), SAMEA5789902 (sample G).

**Impact Statement**

Antimicrobial resistance (AMR) is now recognized as a worldwide public-health issue, and identifying those infections that are resistant to common antibiotics quickly and accurately is a leading priority. The improvement of molecular methods of analysing bacterial DNA, especially whole-genome sequencing (WGS), has raised the possibility of using it as a single assay that can identify the pathogen, antibiotic susceptibility and track transmission. In this study, we compared methods for predicting AMR from bacterial DNA sequences through an inter-laboratory study. This is, to the best of our knowledge, the first study of its kind to blind sets of participants to any contextual information on the samples they were analysing and they were free to choose any analytical pipeline they wanted. This led to variation among the methods used, but also variation in the results reported. Inter-laboratory studies such as these are useful as a precursor to the formal external quality-assurance schemes that come later when assays have been embedded into clinical service. We have shown that although there were discrepancies between results reported, these discrepancies could be traced back to problems such as sequence quality, database choice and user error, all of which can be addressed for WGS to fulfil its potential in clinical settings.

## Introduction

Antimicrobial resistance (AMR) is a major, global, public-health threat, with projections of up to 10 million deaths per annum by 2050 [1]. The World Health Organization’s 2015 Global Action Plan on AMR identified diagnostics as a priority area for combating resistance [2]. Currently, most diagnostic AMR testing is phenotypic antimicrobial-susceptibility testing (AST) and is based on principles dating back to the early 20th century [3]. Molecular testing has facilitated the implementation of PCR assays that target key AMR mutations and genes [4, 5]. However, there remains an unmet need for truly rapid point-of-care AST [6, 7].

Whole-genome sequencing (WGS) is emerging as a routine clinical test that could be used to determine the bacterial species, undertake transmission tracking and identify multiple AMR-associated mutations and genes in a single assay [8–13]. Whilst the initial clinical roll-out of WGS has used cultured bacterial isolates, metagenomics and sequencing direct from clinical samples are future possibilities [14–16]. Resolving the challenges of AMR prediction using WGS for bacteria will provide key advances for the application of metagenomics as a clinical test.

There are currently a wide array of bioinformatics tools and pipelines to predict AMR from WGS data [17]. These have generally been developed by individual researchers and research groups, many with no clinical expertise, and mostly with the same basic principle of matching the input DNA sequence to entries in a reference database of known AMR-associated gene sequences. The testing of pipelines for AMR prediction is typically either performed in-house [18–20] or done ad hoc for specific research [21–24]. Often, these tools are not developed with clinical application or portability in mind. Currently, there are no higher-order reference materials (synthetic references that contain exact components of interest) that are available to validate these tools. Studies have reported good concordance between genotype and phenotype on datasets they have been applied to [9, 22, 25], but rarely address the factors underlying situations where different methods may produce discordant results and how this discordance should be resolved.

Gaining laboratory accreditation is an important, often essential, step for tests in clinical microbiology, but is less advanced for clinical bioinformatics due to its comparatively recent development. Bioinformatic reproducibility studies have been performed for clinically relevant bacterial sequence typing methods [26, 27]. However, while there have been intra-laboratory studies comparing methods of AMR prediction, there have been no comparisons of multiple methods at the inter-laboratory scale. As there is limited evidence of robust, reproducible analyses in bioinformatic prediction of AMR from clinical WGS data, adoption of these methods may be hampered in meeting the necessary accreditation.

This multi-centre study used genomic DNA sequences from clinical carbapenem-resistant organisms, specifically chosen to be of varying quality and complexity, to identify the range of methods used and contributors to discordant AMR predictions. Participants included a mixture of independent individuals and teams using non-commercial AMR prediction pipelines from research groups, hospital laboratories, public-health laboratories and clinical diagnostic companies. The observations made underpin our recommendations for future method developments.

## Methods

### Sample collection and WGS

For the purposes of this study, a panel of ten samples (A-1, A-2, B-1, B-2, C-1, C-2, D, E, F and G) were generated from seven clinical isolates (A, B, C, D, E, F and G). The bacteria were isolated between 2014 and 2017 from stool specimens from patients attending Great Ormond Street Hospital (GOSH), UK, or University Hospital Galway (UHG), Ireland. They represented six clinically relevant bacterial pathogens, including diverse *Enterobacterales* and also *Acinetobacter baumannii *, and contained six distinct families of carbapenemase genes (Table 1).

**Table 1.** Inter-laboratory study sample characteristics

Study ID	Isolate species	Sequencing method	Carbapenemase gene	Median depth of coverage	Comment
A-1	* Klebsiella pneumoniae *	NEBNext Ultra II+NextSeq 150 bp PE	OXA-48-like	190.2×	Exact duplicate of A-2
A-2	* Klebsiella pneumoniae *	NEBNext Ultra II+NextSeq 150 bp PE	OXA-48-like	190.2×	Exact duplicate of A-1
B-1	* Enterobacter cloacae* complex	NEBNext Ultra II+NextSeq 150 bp PE	OXA-48-like	1.4×	Very low coverage duplicate of B-2
B-2	* Enterobacter cloacae* complex	NEBNext Ultra II+NextSeq 150 bp PE	OXA-48-like	142.9×	High coverage duplicate of B-1
C-1	* Klebsiella oxytoca *	Nextera DNA +HiSeq 100 bp PE	OXA-48-like	37.4×	Same original isolate as C-2
C-2	* Klebsiella oxytoca *	NEBNext Ultra II+NextSeq 150 bp PE	OXA-48-like	156.4×	Same original isolate as C-1
D	* Klebsiella pneumoniae *	NEBNext Ultra II+NextSeq 150 bp PE	NDM	83.5×	
E	* Escherichia coli *	Nextera DNA +HiSeq 100 bp PE	IMP	20.6×	
F	* Citrobacter freundii *	NEBNext Ultra II+NextSeq 150 bp PE	VIM	32.5×	
G	* Acinetobacter baumannii *	NEBNext Ultra II+NextSeq 150 bp PE	OXA-23-like and OXA-51-like	22.2×	

PE, Paired end.

Phenotypic AST was performed at UHG and GOSH using the European Committee on Antimicrobial Susceptibility Testing (EUCAST) disc diffusion method (http://www.eucast.org) and meropenem, ertapenem, cefotaxime, amikacin, gentamicin and ciprofloxacin. The isolates were confirmed as carbapenemase producers by PCR at a reference laboratory (Public Health England).

Total genomic DNA was extracted from isolate sweeps on an EZ1 Advanced XL instrument (Qiagen) using DNA Blood 350 µl kits with an additional bead beating step. For eight samples, the NEBNext Ultra II DNA library prep kit (New England Biolabs) and NextSeq (Illumina) 150 bp paired-end sequencing was used. For two samples, the Nextera DNA library prep kit (Illumina) and HiSeq 100 bp paired-end sequencing was used (Table 1). The fastq files were deposited in the European Nucleotide Archive (accession no. PRJEB34513).

### Inter-laboratory study plan

Potential inter-laboratory participants were invited in an individual capacity, both in person and by email, at the meeting ‘Challenges and New Concepts in Antibiotics Research’, March 2018, at Institut Pasteur, France. Fifteen individuals were also emailed directly to participate in the study. From those invited, nine sets of participants agreed to take part in the study. We will refer to these sets as ‘participants’ throughout. These participants were labelled Lab_1 to Lab_9; ‘Lab’ is used as a catch-all term for an individual or team of participants, who came from a mixture of research groups, hospital laboratories, public-health laboratories and clinical diagnostic companies. All participants agreed to take part in a personal capacity using non-commercial pipelines under the condition of anonymity of the results. Each participant was not made aware who the other invited participants were at that stage.

Participants were sent ten paired fastq files (labelled AMRIL_1 to AMRIL_10) and were blinded to their contents. The samples included two exact duplicates A-1 and A-2 (renamed copies of the same fastq files). Two duplicates with different depths of coverage, B-1 and B-2 (sequenced from the same isolate, but with median read depths of 1.4× and 142.9×, respectively). Two samples sequenced from the same isolate, C-1 and C-2 (sequenced in two different laboratories using HiSeq and NextSeq, respectively). The remaining four samples, D, E, F and G, represented diverse bacterial species and carbapenemases.

Participants were asked to report a species identification for each pair of fastq files provided, as well as the presence of all AMR-associated genes present in that sample. They were asked, using the above data, to make a categorical prediction on whether that sample would be resistant to ciprofloxacin, gentamicin, amikacin and cefotaxime. Lastly, participants were asked to provide a description of the analysis pipeline they used.

### Participant analyses

Participants returned results via an Excel spreadsheet (Tables S1–S10, available with the online version of this article). Results were collated for all species identifications and resistant or susceptible predictions from each participant. Collated AMR-associated genes had each name manually checked between each participant to identify minor differences in nomenclature used.

Individual methods are summarized in Table 2. Briefly, all participants used a unique combination of a number of tools to analyse the samples provided and report back results. For species identification, seven participants used a combination of command line tools Kraken [28], Kraken-HLL [29], mash [30], Centrifuge [31] and Kmerid (https://github.com/phe-bioinformatics/kmerid). Four participants also used the web-based tools wgsa (https://pathogen.watch/), blast (https://blast.ncbi.nlm.nih.gov/Blast.cgi) and KmerFinder (https://cge.cbs.dtu.dk/services/KmerFinder/). All participants identified species from raw reads, apart from three participants that used assembled reads (Lab_2, Lab_5 and Lab_8). Lab_3 used both raw reads and assemblies to assign species ID using mash and wgsa, respectively. Six of the nine participating laboratories assembled the raw reads into a draft assembly before identifying AMR-associated genes. Only Lab_4, Lab_7 and Lab_9 used methods that required no assembly of the reads. Of those participants assembling their reads, SPAdes [32] was the most common assembler used, with five participants either using it directly or using one of two wrapper tools that contains it, Unicycler [33] or Shovill (https://github.com/tseemann/shovill). Lab_5 was the only participant to use the assembler A5-MiSeq [34]. Lab_6 was also unique as the only participant to use a commercial bioinformatics platform, Bionumerics (Applied Maths), to perform their analysis. For the identification of AMR-associated genes, ABRicate (https://github.com/tseemann/abricate) and rgi [35] were the most popular tools used, and both take assembled reads as input. The other assembly-based AMR gene identifiers used were c-SSTAR [36] and Resfinder (https://cge.cbs.dtu.dk/services/ResFinder/). Three tools were also used that took raw short reads as input and these were ariba [20], srst2 [37] and Genefinder (https://github.com/phe-bioinformatics/gene_finder). All participants used one or a combination of three AMR databases in their analysis, and these were card [35], Resfinder [18] and arg-annot [38]. The full methods, including command line parameters and software versions, can be found in Supplementary methods.

**Table 2.** Summary of bioinformatic tools used for species identification and detecting AMR by each participant

Method step	Lab_1a*	Lab_1b*	Lab_2	Lab_3	Lab_4	Lab_5	Lab_6	Lab_7	Lab_8	Lab_9	Reference
Species ID	Kraken-HLL	Kraken-HLL	blast	mash and wgsa	Kraken	KmerFinder (assembled contigs)	KmerFinder (raw reads)	Centrifuge	Kraken	Kmerid	[28–31]
Read assembly	Shovill (SPAdes)	Shovill (SPAdes)	SPAdes	Unicycler (SPAdes)	No assembly	A5-MiSeq	Bionumerics	No assembly	Unicycler (SPAdes)	No assembly	[32–34]
AMR identifier	rgi	c-SSTAR	ABRicate	rgi and Resfinder	ariba	rgi	Bionumerics *Escherichia coli* genotyping plugin (blast)	srst2	ABRicate	Genefinder	[18, 20, 35–37]
Reference database	card	Resfinder and arg-annot	card	card and Resfinder	card and arg-annot	card	Resfinder	arg-annot	Resfinder	card and Resfinder (manually curated)	[18, 35, 38]
Sequence identity cut-off	80%	95%	75%	80 % (card) and 90 % (Resfinder)	90%	80%	90%	90%	75%	90%	
Breadth of coverage cut-off	0%	0%	0%	0 % (card) and 80 % (Resfinder)	20%	0%	60%	90%	0%	100%	

*Lab_1 provided two sets of results with two separate methods for AMR detection; these are referred to as Lab_1a and Lab_1b.

## Results

### Bacterial species identification

Four of the nine participants identified all species correctly from WGS data (Table 3). This included the low depth of coverage (1.4×) sample B-1, where we did not expect enough information for a correct call. Species misidentifications of D and B-2 at the genus level by Lab_5 is likely to be a human reporting error, as they correctly identified species in B-1 from a very low read depth. Lab_6 used the same web-based tool for species identification as Lab_5 (Kmerfinder; Center for Genomic Epidemiology), but one error was noted where raw sequence reads were input instead of assembled contiguous sequences (Table 3).

**Table 3.** Species identification for each sample by each participant

Participant	A-1	A-2	B-1	B-2	C-1	C-2	D	E	F	G
Reference	KP	KP	ECl	ECl	KO	KO	KP	EC	CF	AB
Lab_1	KP	KP	ECl	ECl	KO	KO	KP	EC	CF	AB
Lab_2	KP	KP	–	ECl	KO	KO	KP	EC	CF	AB
Lab_3	KP	KP	**Shigella phage SflV**	ECl	KO	KO	KP	EC	*** Citrobacter* sp**.	AB
Lab_4	KP	KP	ECl	ECl	KO	KO	KP	EC	*** Citrobacter* sp**.	AB
Lab_5	KP	KP	ECl	**KP**	KO	KO	**EC**	EC	CF	AB
Lab_6	KP	KP	ECl	ECl	–	KO	*** Klebsiella* sp**.	EC	CF	AB
Lab_7	KP	KP	ECl	ECl	KO	KO	KP	EC	CF	AB
Lab_8	KP	KP	ECl	ECl	KO	KO	KP	EC	CF	AB
Lab_9	KP	KP	ECl	ECl	KO	KO	KP	EC	CF	AB

Missing data represent no results reported. Results highlighted in bold represent discrepancies.

AB, *Acinetobacter baumannii *; CF, *Citrobacter freundii *; EC, *Escherichia coli *; ECl, *Enterobacter cloacae *; KO, *Klebsiella oxytoca *; KP, *Klebsiella pneumoniae *.

### AMR gene identification

**Fig. 1.**
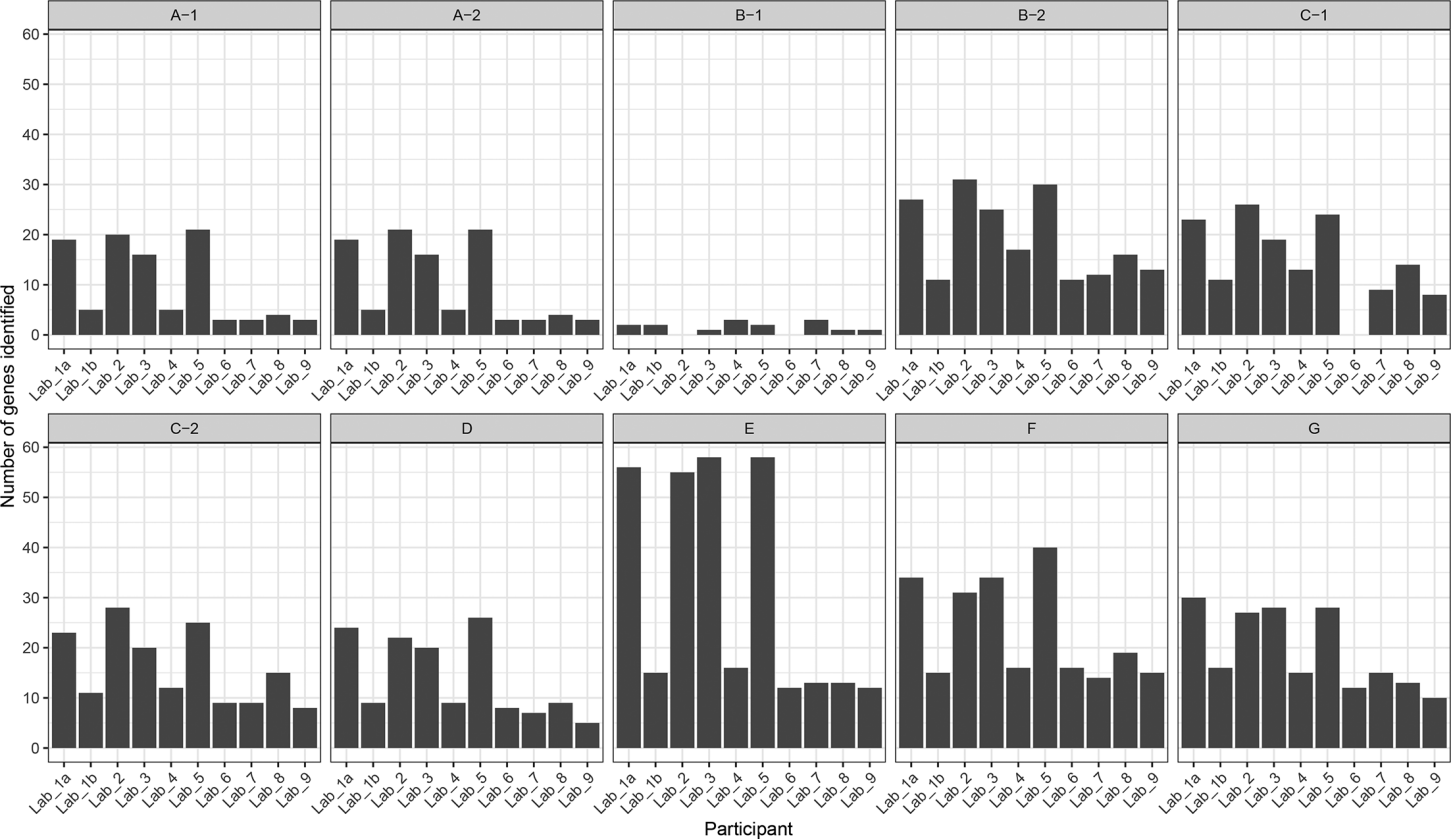
Number of AMR-associated genes identified in each sample by each team of participants.

We compared the number of AMR-associated genes reported by each participant in each sample and found disparities in the total reported (Fig. 1). Lab_1 used two different methodologies for identifying AMR-associated genes; the results are referred to as Lab_1a and Lab_1b. The number of AMR-associated genes reported by each participant was affected by the choice of database used. Lab_1a, Lab_2, Lab_3 and Lab_5 all repeatedly reported the highest number of genes in each sample and all used the Comprehensive Antibiotic Resistance Database (card) as their reference database. This is due to card including many sequences from loosely AMR-associated efflux pump genes that are not found in the other databases. Lab_4 and Lab_9 also used card, but in combination with other databases and selectively reported genes. The number of AMR-associated genes reported by each participant was also found to be associated with sequence identity and breadth of coverage thresholds used to infer a ‘hit’. Both Lab_2 and Lab_8 used the lowest identity and breadth of coverage thresholds (75 % sequence identity and no breadth of coverage threshold), and Lab_2 consistently reported the highest number of AMR genes in each sample. While Lab_8 reported fewer AMR-associated genes than Lab_2, it did use ResFinder as its reference database rather than card, and reported the highest number of genes compared with other participants using the same database.

All isolates included in this study were carbapenem resistant. The reporting of carbapenemase genes from WGS from all participants matched the reference PCR result in 91 % of cases (91/100) (Table 4). Eight of the ten misidentifications occurred in the very low depth of coverage sample B-1, as would be expected. Differences between reported gene variants of *bla*_IMP_ were seen in sample E. Five participants reported *bla*_IMP-1_, whereas the other five reported *bla*_IMP-34_. This discrepancy exactly matched the reference database used with those who reported *bla*_IMP-1_ having used card and those who reported *bla*_IMP-34_ having used either ResFinder or arg-annot. While the sequences for *bla*_IMP-34_ included in each database are identical, the choice of *bla*_IMP-1_ reference sequence included in both databases only share 85 % sequence identity. This is due to card’s *bla*_IMP-1_ reference sequence being isolated from a *Pseudomonas aeruginosa* integron [National Center for Biotechnology Information (NCBI) accession no.: AJ223604] and arg-annot’s reference sequence from an *A. baumannii* integron (NCBI accession no.: HM036079). While there is variation at the nucleotide level, both encode the same IMP-1 enzyme.

**Table 4.** Carbapenemase genes identified for each sample by each participant and the reference laboratory PCR (Ref PCR)

Participant	A-1	A-2	B-1*	B-2	C-1	C-2	D	E	F	G
Ref PCR†	OXA-48-like	OXA-48-like	OXA-48-like	OXA-48-like	OXA-48-like	OXA-48-like	NDM	IMP	VIM	OXA-23-like+OXA-51-like
Lab_1a‡	OXA-48	OXA-48	–	OXA-48	OXA-181	OXA-181	NDM-1	IMP-1	VIM-4	OXA-23+OXA-66
Lab_1b‡	OXA-48	OXA-48	OXA-48	OXA-48	OXA-181	OXA-181	NDM-1	**IMP-34**	VIM-4	OXA-23+OXA-66
Lab_2	OXA-48	OXA-48	–	OXA-48	OXA-181	OXA-181	NDM-1	IMP-1	VIM-4	OXA-23+OXA-66
Lab_3	OXA-48	OXA-48	–	OXA-48	OXA-181	OXA-181	NDM-1	IMP-1	VIM-4	OXA-23+OXA-66
Lab_4	OXA-48	OXA-48	–	OXA-48	OXA-181	OXA-181	NDM-1	**IMP-34+IMP-9**	VIM-4	OXA-23+OXA-66
Lab_5	OXA-48	OXA-48	–	OXA-48	OXA-181	OXA-181	NDM-1	IMP-1	VIM-4	**OXA-23**
Lab_6	OXA-48	OXA-48	–	OXA-48	OXA-181	OXA-181	NDM-1	**IMP-34**	VIM-4	OXA-23+OXA-66
Lab_7	OXA-48	OXA-48	OXA-48	OXA-48	OXA-181	OXA-181	NDM-1	**IMP-34**	VIM-4	OXA-23+OXA-66
Lab_8	OXA-48	OXA-48	–	OXA-48	OXA-181	OXA-181	NDM-1	**IMP-34**	VIM-4	OXA-23+OXA-66
Lab_9	OXA-48	OXA-48	**OXA-405**	OXA-48	OXA-181	OXA-181	NDM-1	IMP-1	VIM-4	OXA-23+OXA-66

*Missing data represent no results reported. Results highlighted in bold represent discrepancies.

†Specific carbapenemase PCR results for each sample.

‡Lab_1 provided different results using two separate methods; these are referred to as Lab_1a and Lab_1b.

**Fig. 2.**
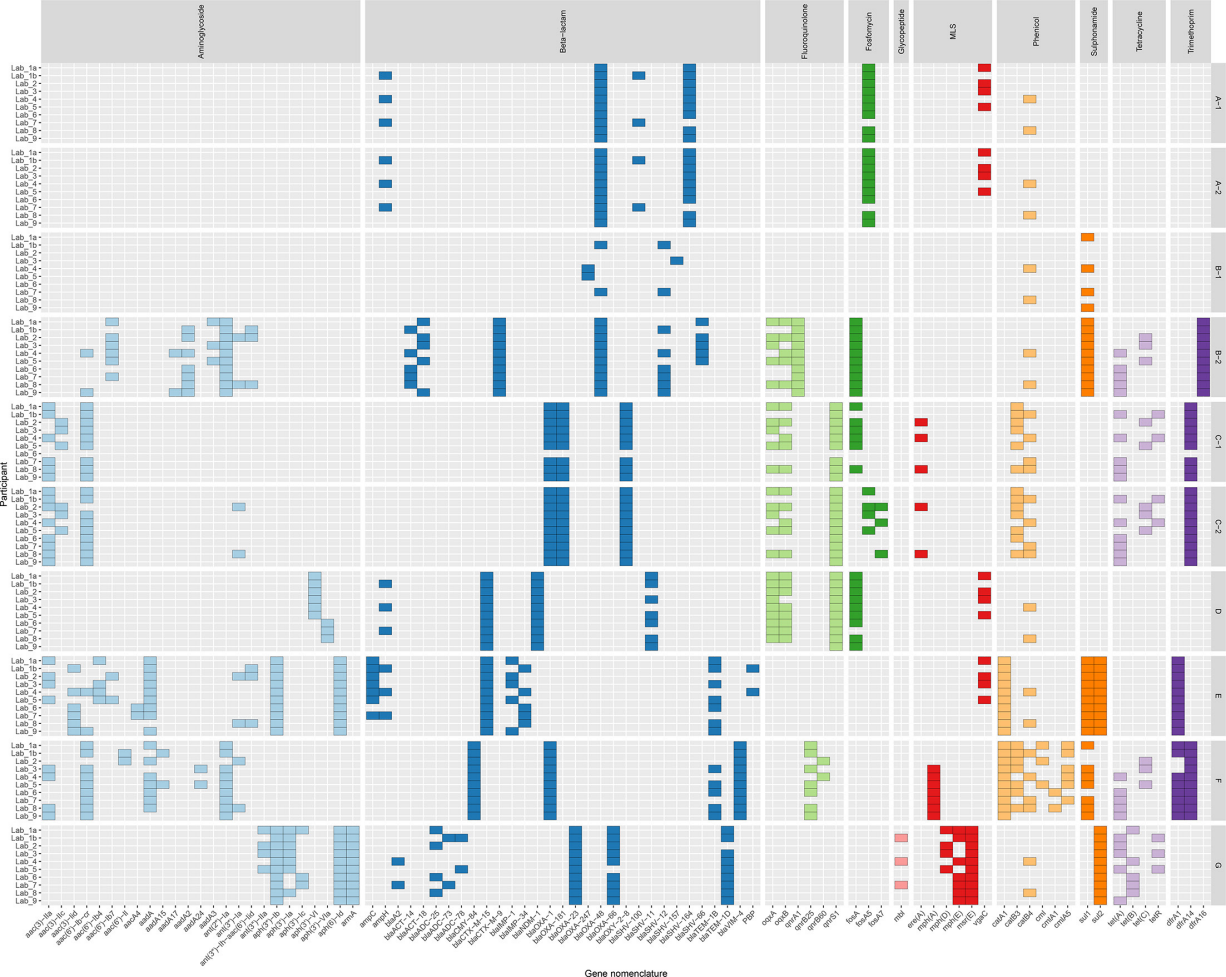
Presence of AMR-associated genes in each sample by each team of participants. Genes are organized and coloured by the class of antibiotics their resistance is associated with. Genes are only shown here if reported by more than one participant and if they were present in more than one reference database used. MLS, Macrolide lincosamide and streptogramin.

We compared all AMR-associated genes identified by each participant in each sample. As previously noted, the largest discrepancies were the 55 efflux pump gene sequences that were present only in card (Fig. S1). To understand the other factors influencing discordant reporting, we removed these genes that were only present in one database from our comparisons (Fig. 2). A pairwise comparison between all participants found that two sets of participants only reported the exact same genes within a sample in 2 % (18/900) of cases. Fourteen of these cases occurred when analysing the two identical samples (A-1 and A-2; Fig. 2). Although there was little agreement between participants for genes identified in A-1 and A-2, there was complete within-participant concordance across both samples, exhibiting reproducibility within each analysis pipeline. No two participants reported the exact same combination of gene variants in samples B-2, C-1, D, F and G. There were many clear examples where participants assigned different gene variants to the same sequence data where the reference sequences only differed by a few single nucleotides. This can be seen in Fig. 2 amongst samples that contained tetracycline-resistance genes [*tet(A*), *tet(B*) and *tet(C*)], some aminoglycoside modifying enzyme gene variants [*aac(3)-IIa* and *aac(3)-IIc*] and *β*-lactamases (*bla*_ACT-14_ and *bla*_ACT-18_). We also observed differences between the same participants analysing samples from the same original isolate. Due to the very low read depth, the genes reported in B-1 bore little resemblance to B-2 across all participant results. However, even in the samples from the same isolates with sufficient sequencing depth (C-1 and C-2), we observed differences in the genes identified in four out of nine participants. This suggests that resequencing, and even small increases in read length and quality, can produce variation in results. It is worth noting that all but one of these differences were additional genes identified in C-2, which had a higher read depth than B-2 (156 vs 37× median read depth). The additional genes in C-2 included *ant(3′′)−Ia* (Lab_2 and Lab_8), *fosA7* (Lab_2 and Lab_8) and *tet(C*) (Lab_3), but the reported reference breadth of coverage of *ant(3′′)−Ia* and *fosA7* was low (17 and 75 %, respectively) and the sequence similarity between the purported *tet(C*) sequence and the reference was also low (75%). We also found no systematic differences in genes present or absent between those participants that used tools that required assembly of short reads first and those that took unassembled short reads as input (Lab_4, Lab_7 and Lab_9, ariba, srst2 and Genefinder, respectively).

### Phenotypic and genotypic resistance concordance

**Fig. 3.**
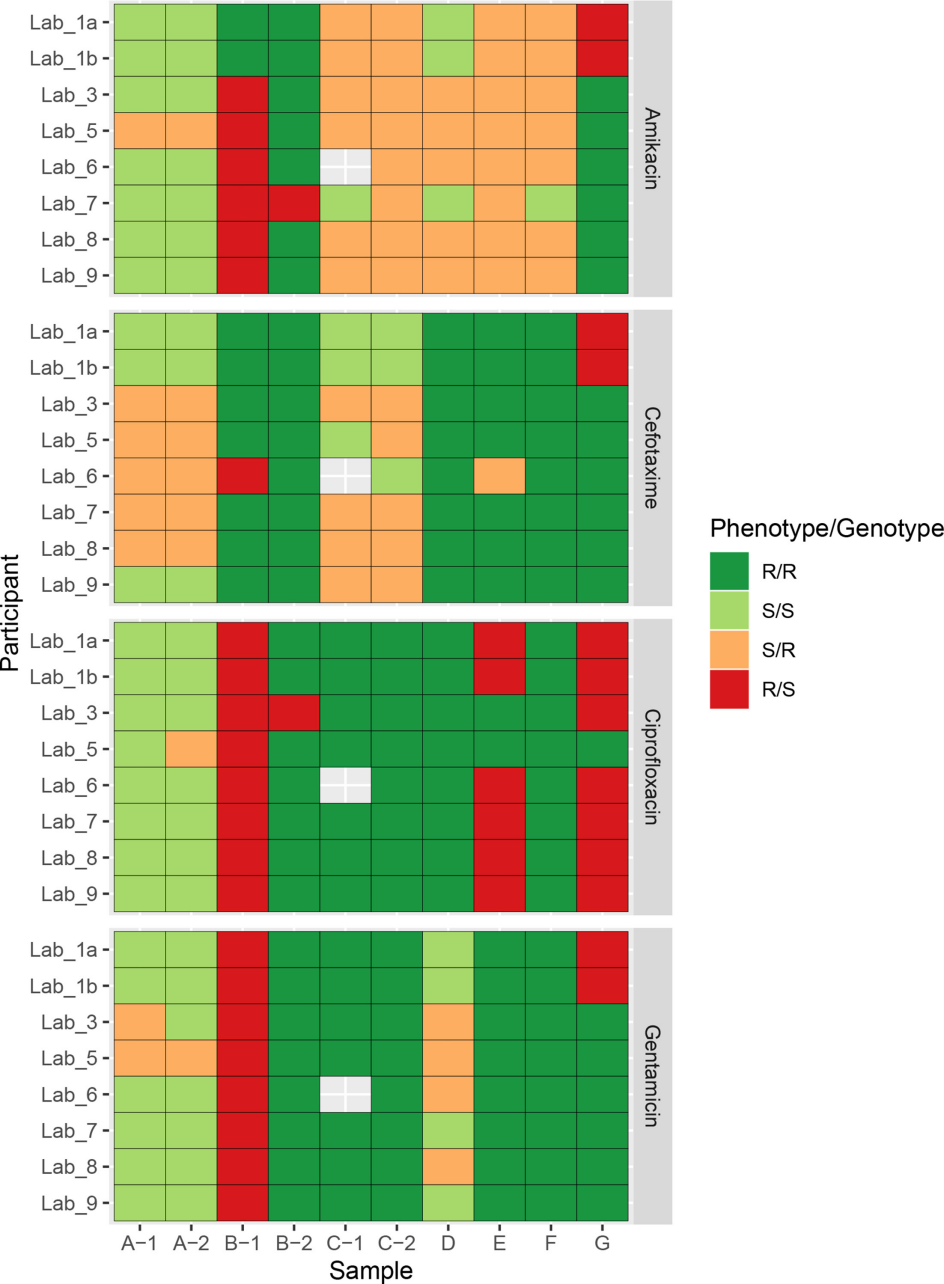
Concordance between phenotypic AST result and the genotypic prediction from WGS data. Results are presented separately for each participant, sample and antibiotic. Each tile is coloured based on whether both the resistant phenotype and genotype agreed (R/R); both phenotype and genotype predicted sensitive (S/S); major errors where the phenotype was sensitive, but the genotype was resistant (S/R); and very major errors where the phenotype was resistant, but the genotype was sensitive (R/S). Missing cells represent a result not reported.

Given the differences in the AMR-associated genes identified in the samples by each participant, we also compared predictions of antibiotic resistance to phenotypic AST results and each other. Two participants (Lab_2 and Lab_4) did not submit any results for phenotypic resistance prediction, so were not included in the subsequent analysis. A pairwise comparison between genotypic prediction results reported by all participants, on all antibiotics and samples, showed an overall consensus of 79 % (864/1092, Fig. 3). This varied depending on the antibiotic tested with the highest pairwise reporting consensus of 88 % (240/273) between participants for ciprofloxacin and the lowest pairwise reporting consensus of 72 % (197/273) for cefotaxime, which could be understandable given the different complexities of the resistance mechanisms involved. When we compared results from each participant with the phenotypic AST results, we found an overall sensitivity of 76 % and specificity of 50 %. The overall number of false positives was 64/316 (20 %) and the overall number of false negatives was 44/316 (14 %). Lab_5 had the highest number of false positives (14/40) and lowest number of false negatives (3/40), whereas Lab_1 had the lowest number of false positives (4/40) but the highest number of false negatives (7/40). Broken down by antibiotic, the highest consensus between phenotype and genotype was gentamicin (78%, 62/79) and the lowest amikacin (43 % 34/79). As expected, there was little agreement between predictions within the very low read depth sample (B-1) and most participants predicted a susceptible isolate due to missing data when in fact it was resistant by phenotypic AST. However, when analysing the same isolate at an appropriate higher read depth (B-2), there was near perfect concordance between participant reported genotypes and the resistance phenotype, with only two discrepant results reported by Lab_3 (ciprofloxacin) and Lab_7 (amikacin). Lab_3 also reported different results between the two identical samples (A-1 and A-2), where A-1 was reported as resistant and A-2 was reported as sensitive. As there were no differences in the gene content reported in either sample by this participant (Fig. 2), this is likely to be due to a human reporting error. We also identified a single discrepancy between amikacin resistance predicted by Lab_7 between samples C-1 and C-2, which both were sequenced from the same isolate. C-1 was reported as sensitive but C-2 was reported as resistant, and the phenotypic AST result was sensitive; however, there was no difference in the reported gene content in both samples by Lab_7, so it is also another likely human reporting error. Excluding the extremely low depth sample, B-1, there were only 2/30 cases where no laboratory correctly predicted the phenotypic AST result. Both of these results were an incorrect resistance prediction for amikacin in C-2 and E, but as noted earlier the prediction from Lab_7 for C-2 was likely human error.

## Discussion

In this study, we have shown that participants using different choices of bioinformatics pipelines reported different AMR-associated gene variants when given identical mixed quality bacterial isolate WGS datasets. This led to differences in the reporting of predicted resistance phenotypes. We observed good concordance for genotypic-resistance predictions between participants, but poor concordance with phenotypic AST results. A similar trend has previously been seen in a study of *Staphylococcus aureus* genomes [39]. Concordance in phenotype prediction differed for different antibiotic classes. Good concordance was seen comparing WGS with AST results for gentamicin, but for amikacin concordance was poor. This may be due to the fact that amikacin is not affected by the action of most aminoglycoside-modifying enzymes [40]. Previous studies predicting antimicrobial susceptibility from WGS data have reported sensitivities of 96 and 99 % against phenotypic AST as a benchmark [21, 22], compared with an overall sensitivity of 76 % in this inter-laboratory study. It should be noted, however, that some of the data used in this study were purposefully very low quality, with some of the clinical isolates deliberately chosen to be difficult to characterize. Similar mixed quality data tested using current clinical AST phenotyping may also result in equivalent discrepancies. However, our aim here was to document the range of bioinformatics approaches being used and identify plausible contributors to discordant results reported between participants working on the same data, in order to provide useful recommendations and direct future work.

We identified three stages of analysis that contributed to discrepancies in predictions: the quality of the sequence data used, the bioinformatic methods (choice of database or software used) and the interpretation of those results. Where single gene calling is required (e.g. presence of a carbapenemase), results are mainly affected by sequence quality. However, once multiple genes are involved, all three analytical issues become important. We found the largest contributors to discrepant results between the gene variants reported in each sample and the phenotypic resistance predictions were the sample sequence quality, read depth and the choice of reference resistance-gene database. Samples must be sequenced to a sufficient depth as well as sufficient breadth of coverage for the expected size of the genome, usually inferred by mapping to a suitable reference genome, of at least above 90 %. Based on our own experience and these results, we recommend 30× depth as a lower limit. This also tends to be a default setting for many read assembly tools, but generally most samples should have a higher depth of coverage than this for meaningful prediction. Some participants did flag that they would not normally analyse the low depth of coverage samples (<30×, samples B-1, E and G) and if those samples are excluded from this analysis sensitivity in comparison to phenotypic AST rises from 76 to 98 %. This is highly encouraging as it suggests that as long as the sequence data produced is of sufficient depth and quality (e.g. current Illumina error rates) genotypic prediction of resistance phenotype can be comparable to AST. However, we also note that many sets of participants provided little information on their employment of quality control and filtering steps. Our results, therefore, suggest an increased emphasis on data quality control is highly relevant to improving sensitivity. Conversely, we have observed the choice of sequencer and DNA library preparation method has a small effect on closely related gene variants, but little discernible effect on the inference of resistance phenotype.

Some participants ran the same set of read data against different reference databases and merged the results, which led to different gene variants being reported at the same loci. In practice, different variants of the same gene may not always result in a different clinically relevant phenotype. However, we also found reference sequences in different databases for same gene variant can differ by 15 % nucleotide identity (*bla*_IMP-1_ in card and arg-annot). If precise identification of gene variants is required, we would strongly recommend avoiding this, as it effectively leads to ‘double-dipping’ using the same reads. Multiple reference databases could be used, but after screening for reads that have already been assigned a hit against one of the databases. This would avoid multiple different genes reported at the same genomic loci. However, it would be better to merge the different reference databases and remove the redundant sequences before comparisons are made against the test data. Sequence identity, and to lesser extent breadth of coverage cut-offs, should be kept high when comparing test data to a reference database. Based on this study, we would recommend using a sequence identity cut-off of at least 90%, in combination with an up to date curated reference resistance-gene database. Although lowering of these thresholds does identify more candidate genes within a sample, many were false negatives; thus, not improving concordance with phenotypic AST results in this study.

There is an overwhelming need for a standardized, centralized database that integrates the current knowledge base for linking genotype with resistance phenotype and is not linked to a single research group, as previously suggested [10]. There is also a growing need regarding computational reproducibility [41, 42]. This would deal with many of the issues we have raised, such as which sequences to include and what gene nomenclature to use. With strict version control, such a resource would allow greater integration of results and be an invaluable tool for larger epidemiological studies. Currently, databases are being built for organisms such as for *Mycobacterium tuberculosis *, though this is a less challenging organism for genotype–phenotype predictions due to it being highly clonal and lacking an accessory genome [43, 44]. A recent publication of a new protein-based database also obtained high concordance (98.4%) between genotype and phenotype for four food-borne pathogens [45]. However, for other clinically relevant organisms there are limited resources.

Participants in this study included a mixture of individuals and teams involved in AMR prediction in a variety of settings. A potential criticism is that we did not restrict these settings to those routinely predicting AMR phenotype for clinical use, meaning that some participants were attempting analyses they did not usually perform. However, the fact that AMR phenotype prediction from WGS is not yet routine in most clinical laboratories was the very reason for undertaking this study. Clinical laboratories at the moment do not have the tools or knowledge to make good phenotypic resistance calls from genotypic data. This is evident from the fact that two participants in this study did not report any phenotypic resistance predictions as they felt they could find no valid method for doing so. At this point in time, many research laboratories use these methods to track specific resistance genes or one specific resistance mechanism, rather than building tools for the broad detection of AMR in bacteria for clinical purposes. We found in this study that there was particularly low concordance between participants reporting sensitive isolates compared with phenotypic AST. The problem with the inference of phenotype from genotype is that the information either is not known at all or is expert knowledge restricted to single laboratories working on specific bacteria. In addition to this, although the identification of the presence of genes is performed in a systematic way, the prediction of resistance is still performed in an ad hoc manner by scientists and, therefore, subject to user error given the same set of genes. Once again, *M. tuberculosis* is providing the first example of the need for a defined decision tree when working from the presence of genes or gene variants to the prediction of phenotypic drug resistance [46]. Interpretation and reporting of this genotypic data will need to be subjected to the same level of scrutiny as current tests if it is to form part of an accredited laboratory service within the healthcare service.

A limitation of this study is that we focused on the use of short-read sequence data, which produces sequences far shorter than the length of genes being identified. However, we feel this is more reflective of the WGS data that is more routinely generated in clinical laboratories at this point in time. If these short reads need to be assembled into longer contiguous sequences, we found it essential to use an actively developed short-read assembler such as SPAdes (http://cab.spbu.ru/software/spades/). Web-hosted tools that provide a ‘black box’ solution to assembly and identifying resistance from uploaded WGS data should be avoided if possible, because of the lack of interpretability. Tools are needed that are open source, designed for clinical purpose and can be subjected to thorough troubleshooting when erroneous results arise [47]. To this end, permanently employed bioinformaticians are required, who can provide expert interpretation of the results and update approaches as necessary. In this study, tools that either require assembled contigs (ABRicate) and those that take unassembled short reads (srst2 and ariba) were capable of producing very similar results with no notable effects alone on the predication of phenotypic resistance. This holds promise for rapid phenotypic predictions, as genome assembly is one of the largest bottlenecks in computational analysis time.

Other limitations of this study include our focus on acquired genes rather than point mutations or many of the other resistance mechanisms found in bacteria (e.g. target site modifications and efflux pumps). We also only required reporting on categorical resistance predictions. Furthermore, because our focus was on WGS, and although we validated AST at two independent laboratories, we did not investigate potential variability and discordance in phenotypic prediction. More work needs to be done on the prediction of minimum inhibitory concentrations (MICs) from WGS data before it can be implemented in laboratories. This will be aided by more systematic reporting of accompanying MIC data when making WGS data available.

We have outlined recommendations for improving the current state of prediction of AMR from WGS data. Some of these recommendations, such as a standardized database and better dissemination of phenotype/genotype relationships, cannot be implemented immediately. However, current pipelines can be improved right now by robust quality control of starting sequence reads to make sure that the genome breadth of coverage is high (>90 %) and that there is sufficient depth of coverage (>30×). We also recommended that running the same sequence read data set against multiple databases should be avoided due to the erroneous results, and that sequence identity between the predicted and reference AMR genes should be higher than 90 % to avoid non-specific hits. We found little difference between the results of participants depending on what reference database they chose to use, between which Illumina short-read sequencer was used and whether they used assembly or assembly-free methods.

In conclusion, we have identified some of the current contributors to discrepancies in predicting AMR-associated genes and phenotypes from bacterial isolate WGS data. We have provided recommendations for improving the current reporting of results. Despite its clear potential, even after accounting for poor sequence data, we found that the current public methods, in particular databases, are not adequate ‘off-the-shelf’ tools for the prediction of AMR from bacterial WGS data as a universal clinical test at this point in time.

## Data bibliography

1. Doyle, RM. Sequence read files for all samples used in this study have been deposited in the European Nucleotide Archive under the project accession number PRJEB34513 (2019).

## Supplementary Data

Supplementary material 1

Supplementary material 2
